# Randomized, active-controlled, comparative phase 3 efficacy and safety equivalence trial of Ovaleap® (recombinant human follicle-stimulating hormone) in infertile women using assisted reproduction technology (ART)

**DOI:** 10.1186/s12958-015-0135-8

**Published:** 2016-01-06

**Authors:** Thomas Strowitzki, Waldemar Kuczynski, Arnd Mueller, Peter Bias

**Affiliations:** Department of Gynecological Endocrinology and Reproductive Medicine, University of Heidelberg, Im Neuenheimer Feld 440, 69120 Heidelberg, Germany; Centre for Reproductive Medicine, Cryobank, 15879 Bialystok, Poland; Department of Gynaecology and Gynaecological Oncology, Medical University, 15089 Bialystok, Poland; Merckle GmbH, A Member of The Teva Group, 89079 Ulm, Germany

**Keywords:** Ovaleap, Follitropin alfa, Gonal-f, Infertility, Assisted reproductive technology (ART)

## Abstract

**Background:**

Pharmacokinetic studies with XM17 (Ovaleap®), a recombinant human follicle-stimulating hormone (r-hFSH, follitropin alfa), have demonstrated good safety and tolerability in healthy women whose endogenous FSH levels were down-regulated with a long agonist protocol. In these studies, Ovaleap® pharmacokinetics were dose-proportional and bioequivalent to the reference follitropin alfa product (Gonal-f®). The objective of the present study is to determine whether Ovaleap® is equivalent to Gonal-f® with respect to the number of oocytes retrieved in infertile but ovulatory women undergoing assisted reproductive technology (ART) therapy.

**Methods:**

This multinational, multicenter, randomized (1:1), active-controlled, assessor-blind, comparative study included infertile normally gonadotrophic women 18 to 37 years old with a body mass index of 18 to 29 kg/m^2^ and regular menstrual cycles of 21 to 35 days undergoing ART therapy. During a 5-day fixed-dose phase, women received 150 IU/day of Ovaleap® (n = 153) or Gonal-f® (n = 146), followed by an up to 15-day dose-adaptation phase during which doses could be adjusted every 3 to 5 days, up to a maximum of 450 IU/day. Ovaleap® was to be deemed equivalent to Gonal-f® if the two-sided 0.95 confidence interval (CI) for the difference in the number of oocytes retrieved fell within the equivalence range of ±3 oocytes.

**Results:**

Similar numbers of oocytes were retrieved in the 2 treatment groups. The mean ± SD number of oocytes retrieved was 12.2 ± 6.7 in the Ovaleap® group and 12.1 ± 6.7 in the Gonal-f® group (intent-to-treat [ITT] population). Regression analysis estimated a mean difference of 0.03 oocytes between the treatment groups (95 % CI: −0.76-0.82), which was well within the prespecified equivalence range of ±3 oocytes. Ovaleap® and Gonal-f® showed favorable and comparable safety profiles, with no unexpected safety findings.

**Conclusions:**

Ovaleap® has shown the same efficacy and safety as Gonal-f® for stimulation of follicular development in infertile women (up to 37 years of age) who are undergoing ART therapy.

**Trial Registration:**

EudraCT: 2009-017674-20. Current controlled trials: ISRCTN74772901. Date of trial registration: 19 March 2010.

## Background

Exogenous gonadotropins, including follicle-stimulating hormone (FSH), are often used to treat infertility by inducing ovulation or controlled ovarian stimulation in the context of assisted reproductive technology (ART). Available FSH products include urinary-derived human menopausal gonadotropin (hMG), purified urinary FSH, highly purified urinary FSH, and recombinant human FSH (r-hFSH) [1, 2].

r-hFSH was developed, in part, to increase FSH production independent of urine collection, to ensure greater availability, and to reduce the variability due to the inherent inconsistency of the starting materials [3]. r-hFSH also offers the potential additional benefit of a reduced risk of immunological reactions due to impurities [3, 4].

Marketed r-hFSH products available in Europe include follitropin alfa (Gonal-f® and Bemfola®), follitropin beta (Puregon®), and a long-acting FSH, corifollitropin alfa (Elonva®).

Ovaleap® (follitropin alfa) is an r-hFSH manufactured in Chinese hamster ovary (CHO) cells. It was developed as a biosimilar to Gonal-f® as recommended by the European Union in their guidelines for the clinical development of biological medicinal products that contain r-hFSH [5], and was approved by the European Medicines Association as a biosimilar in 2013. Biosimilar formulations represent products with demonstrated similarity in physicochemical characteristics, efficacy, and safety to an approved product in comprehensive comparability studies [6]. Ovaleap® is primarily intended for use in controlled ovarian stimulation in women undergoing ART procedures and for the treatment of anovulation. It is also indicated to treat women with severe FSH or luteinizing hormone deficiency and to stimulate spermatogenesis in men. The amino acid sequence of Ovaleap® is identical to that of the follitropins currently registered in Europe, and its tertiary structure is also similar.

Two pharmacokinetic studies of Ovaleap® in healthy female subjects have provided preliminary evidence of its potential utility. In a dose-response study, Ovaleap® administered in single subcutaneous (SC) doses up to 300 IU to healthy pituitary down-regulated women (*N* = 40), demonstrated dose-proportionality and was well tolerated [7, 8]. A second study demonstrated the bioequivalence of 300 IU of Ovaleap® and 300 IU of Gonal-f® as assessed by peak plasma concentration (C_max_) and area under the concentration vs time curve (AUC_0-t_) [9]. In both of these pharmacokinetic studies, Ovaleap® was safe and well tolerated.

The primary objective of this phase 3 patient-study was to evaluate the clinical efficacy of Ovaleap® compared with Gonal-f® in terms of the number of oocytes retrieved and safety in infertile ovulatory women undergoing ART therapy. The number of oocytes retrieved is the primary endpoint recommended by the European Medicines Agency for studies evaluating the clinical comparability of Gonal-f® and the similar formulation [5]. Moreover, historical data have demonstrated the strong association between the number of retrieved eggs and live birth rates [10]. Other efficacy parameters were also compared, as were safety and tolerability.

## Methods

This was a multinational, randomized, active-controlled, assessor (ie, investigator and embryologist)-blinded, parallel-group patient study conducted between March 2010 and July 2011 in 20 centers in 5 countries (Belgium, Czech Republic, Germany, Hungary, and Poland). The study protocol and informed consent documents were approved by the relevant independent ethics committees. The study was conducted in accordance with the Good Clinical Practice Consolidated Guideline, according to the International Conference on Harmonisation and the Declaration of Helsinki (1996).

### Participants

Women aged 18 to 37 years were eligible for inclusion in the study if they were infertile but otherwise healthy, were normally gonadotrophic, had 2 confirmed normal ovaries, and were undergoing controlled ovarian stimulation with ART therapy. Women also must have had regular menstrual cycles of 21 to 35 days; body mass index (BMI) between 18 and 29 kg/m^2^; and basal FSH, estradiol, prolactin, and thyroid-stimulating hormone concentrations in the normal range.

Women who had more than 2 previously completed consecutive unsuccessful in vitro fertilization (IVF) cycles or more than 3 miscarriages were excluded from the trial; as were women with a history of severe ovarian hyperstimulation syndrome (OHSS) as defined by Papanikolaou and colleagues [11], primary ovarian failure or being categorized as poor responders to ovarian stimulation, or hypersensitivity or allergy to rFSH preparations. Also excluded from the trial were women who had any significant cardiovascular, pulmonary, neurologic, endocrine, hepatic, or renal disease; or neoplasm or a history of chemotherapy or radiation therapy; or who had used clomiphene or gonadotropins within 30 days prior to enrollment.

To become eligible for inclusion in the study, participants were required to give written informed consent. Study participants were free to discontinue at any time.

### Pituitary down-regulation, ovarian stimulation, and oocyte retrieval

**Fig. 1:**
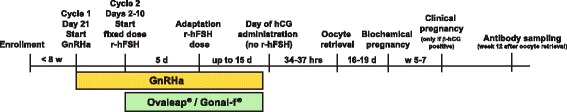
Study design. GnRHa, gonadotropin-releasing hormone agonist; r-hFSH, recombinant human follicle stimulating hormone; hCG, human chorionic gonadotropin; ẞ-hCG, beta-human chorionic gonadotropin; w, weeks; d, days; hrs, hours

Starting at approximately Cycle Day 21, eligible patients received the gonadotropin-releasing hormone agonist buserelin acetate (Metrelef®, Ferring Arzneimittel GmbH, Kiel, Germany) to down-regulate endogenous FSH, as shown in Fig. 1.

After confirmation of down-regulation, women with serum estradiol < 50 pg/mL and a negative pregnancy test were randomly assigned (1:1) to receive either Ovaleap® or Gonal-f® through an interactive voice response system initiated by study personnel. The randomization scheme was computer-generated and stratified by study center using a block size of 2. Women with ovarian cysts > 10 mm, verified by ultrasound after down-regulation, could not be randomized to study treatment and were excluded from the trial. Investigators received no information about treatment allocation and the size of the randomization blocks was not disclosed in the protocol or to the study centers. The investigators and embryologists were kept blinded and performed all assessments without knowledge of treatment.

Ovaleap® (Merckle Biotec GmbH, Ulm, Germany) was supplied in glass cartridges containing 900 IU in 1.5 mL solution to be administered from a reusable semi-automated pen device. Gonal-f® (Merck Serono S.p.A., Modugno, Italy) contained 900 IU in 1.5 mL solution to be administered using a prefilled pen. Because the drugs were administered using two different and unique pen devices, a double-blind study design was not feasible. However, a single-blind design was maintained through the use of a “drug administrator” (eg, a physician or nurse) who instructed the patient how to use the study drug, but who was not involved in any study assessments. The drug administrator also instructed the patient not to disclose her assigned study drug (Ovaleap® or Gonal-f®) to the investigator and the embryologist.

Eligible patients were treated for 1 stimulation cycle with Ovaleap® or Gonal-f® as stimulating agent using a long GnRH agonist protocol. Patients received SC injections in fixed doses of 150 IU of Ovaleap® or Gonal-f® daily for the first 5 days. The first dose was injected by the drug administrator at the study center; subsequent doses were self-administered by the patient. To achieve adequate follicular development, doses could be adjusted (up or down) at the investigators’ discretion (based on their assessment of follicle count, size, and appearance) after Day 5 every 3 to 5 days through Day 20 in increments of 37.5 IU (but no more than 150 IU) to a maximum of 450 IU/day, based on serum estradiol levels and ultrasound examinations.

Women who had at least 3 follicles with a mean diameter of 17 mm, as assessed using transvaginal ultrasound, and estradiol levels below 5500 pg/mL then received human chorionic gonadotropin (hCG; Ovitrelle®, Merck Serono S.p.A., Modugno, Italy) to induce follicular maturation and trigger ovulation.

Oocyte retrieval took place 34 to 37 h after hCG administration. Luteal support was provided after oocyte retrieval at the investigators’ discretion. Evaluation of biochemical pregnancy (defined as a positive β-hCG test) occurred 16 to 19 days after oocyte retrieval, while evaluation of clinical pregnancy (defined as gestational sac with fetal heart activity) occurred 5 to 7 weeks post-oocyte retrieval.

### Outcome measures

The primary endpoint was the number of oocytes retrieved. Secondary endpoints included total r-hFSH dose, number of patients needing dose adaptations, duration of dosing, number of follicles, serum estradiol concentration, and endometrial thickness prior to dose adaptation (Stimulation Day 6) and on the day of hCG injection, and cancellation rate prior to oocyte retrieval. After oocyte retrieval and fertilization, fertilization outcome was assessed and recorded using the following categories: without pronucleus (PN), 1 PN, 2 PN, 3 or more PN, and other. The quality of all 2 PN oocytes (zygotes) was then evaluated using the zygote scoring system of Scott et al. [12]. Number of embryos obtained, transferred, and cryopreserved; biochemical and clinical pregnancy rate; and take-home baby rate (defined as the percentage of randomized patients with live births) were also assessed. Patients with clinical pregnancies were followed until birth for any complications during pregnancy and delivery.

Safety and tolerability were monitored by recording treatment-emergent adverse events (TEAEs), including OHSS; results of laboratory tests, including assessments of hematology, clinical chemistry, hormone levels, and immunogenicity (antibody levels); electrocardiograms (ECGs); vital signs; body weight measurements; local and overall tolerability, physical examinations, and patient satisfaction with the pen device.

Anti-FSH antibodies were assessed in all randomized patients at 3 time points: baseline, day of oocyte retrieval, and 3 months after the last administration of Ovaleap® or Gonal-f®. Serum samples were assessed for the presence of anti-FSH antibodies using validated assays at 2 central laboratories. The initial anti-drug antibodies assay was revised for sensitive detection of anti-Ovaleap® and anti-N-glycolylneuraminic acid (anti-Neu5Gc) antibodies in human serum samples. The sensitivity of the new assay increased by about 10-fold.

Patients evaluated injection-site pain after each injection using a numerical scale ranging from 0 (no pain at all) to 10 (the most severe pain). They also recorded the presence and intensity of injection-site reactions, such as redness, bruising, swelling, burning, or skin irritation. In addition, patients rated the convenience of the pen using a 3-point rating scale, as well as their satisfaction with the administration of r-hFSH using a pen by completing a questionnaire based on that used by Somkuti et al. [13].

### Statistical analysis

The aim of the study was to evaluate equivalence of Ovaleap® compared with Gonal-f® with respect to the primary efficacy endpoint, the number of oocytes retrieved. A sample size of 124 patients per group was determined to be necessary to achieve 90 % power (a two-sided level of α = 0.05) for rejecting the null hypothesis that Ovaleap® is different from Gonal-f®. Ovaleap® was to be considered clinically equivalent to Gonal-f® if the difference in the mean number of oocytes retrieved between the 2 groups was ≤ 3 (primary endpoint). The prespecified margin of 3 oocytes has been used in previous equivalence trials of Gonal-f® [14, 15]. The anticipated difference in the expected mean number of oocytes was ≤ 0.5 with a common standard deviation of 6 oocytes. Assuming that about 10 % of patients would not be eligible for the analysis of the primary endpoint due to protocol violations or dropouts, 140 patients per treatment group were to be included in the trial.

A zero-inflated Poisson regression (ZIP) was used to assess the primary endpoint, with treatment and country as fixed factors and age as a covariate. The primary endpoint was evaluated in all randomized patients (intent-to-treat [ITT] population) as well as in those who did not have any major protocol violations (according-to-protocol [ATP] population). The safety analysis included all randomized patients who received at least 1 dose of r-hFSH (Ovaleap® or Gonal-f®). A stratified post-hoc analysis of clinical and ongoing pregnancy rates by baseline and post-baseline characteristics was also performed.

Secondary endpoints, presented only for the ITT population, were assessed using descriptive statistics (eg, mean ± SD, median, and range). Stratified odds ratios and related *P*-values on secondary endpoints were calculated using Mantel-Haenszel tests. Since resultant *P*-values were regarded as supportive only, no adjustment for multiple testing was made.

## Results

### Subject disposition and baseline characteristics

**Fig. 2:**
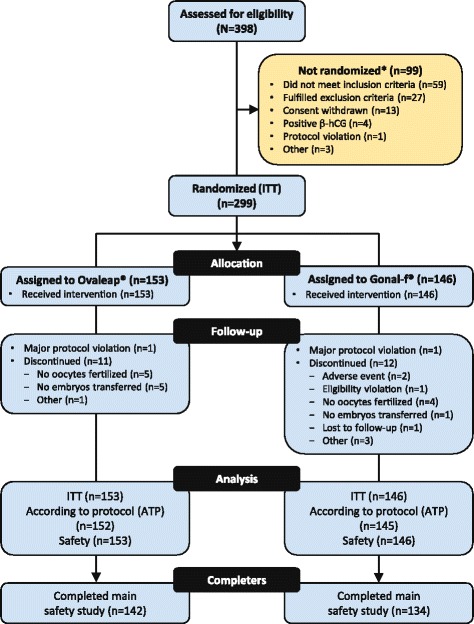
Patient disposition. ITT, intent-to-treat. *Subjects could be excluded for multiple reasons. The 2 protocol violations post-allocation involved patients whose dosing was adapted during the fixed-dose phase

Of the 398 women screened, 299 were randomized (ITT population) to receive either Ovaleap® (*n* = 153) or Gonal-f® (*n* = 146), as shown in Fig. 2. The number of patients randomized per study center ranged from 1 to 2 at study centers in Belgium, 6 to 17 at study centers in the Czech Republic, 3 to 16 at study centers in Germany, 36 to 41 at study centers in Hungary, and 18 to 30 at study centers in Poland (Table 1). The ATP population (n = 297) differed only slightly from the ITT population in that 2 patients (1 per group) in the ITT population underwent dose adaptation during the fixed-dose period, which was considered a major protocol violation. Demographic and baseline characteristics—including age, weight, BMI, smoking, and alcohol consumption, as well as reasons for, and durations of, infertility—were comparable between treatment groups (Table 2).

**Table 1:** Distribution of patients by country (ITT population)

Country	Ovaleap® (*n* = 153)	Gonal-f® (*n* = 146)	Total (*N* = 299)
Belgium + Germany^a^	35	30	65
Czech Republic	30	28	58
Hungary	39	38	77
Poland	49	50	99

^a^3 patients were randomized in Belgium: 1 to Gonal-f® and 2 to Ovaleap®

**Table 2:** Baseline demographics and patient characteristics (ITT population)

Characteristic	Ovaleap® (*n* = 153)	Gonal-f® (*n* = 146)
Age, years, mean (SD)	31.6 (3.1)	31.7 (3.2)
Age, *n* (%)		
< 30 years	35 (22.9)	36 (24.7)
30 to 34 years	93 (60.8)	75 (51.4)
> 34 years	25 (16.3)	35 (24.0)
Weight, kg, mean (SD)	63.8 (10.2)	63.1 (9.2)
BMI, kg/m^2^, mean (SD)	22.8 (2.9)	22.6 (2.9)
Smoker, n (%)	18 (11.8)	19 (13.0)
Alcohol consumption, *n* (%)	33 (21.6)	23 (15.8)
Duration of infertility, in months at baseline, mean (SD)	43.6 (26.2)	46.6 (29.0)
Causes of infertility^a^, *n* (%)		
Male factor	86 (56.2)	77 (52.7)
Idiopathic	39 (25.5)	41 (28.1)
Tubal factor	26 (17.0)	30 (20.5)
Endometriosis	10 (6.5)	10 (6.8)
Other	6 (3.9)	10 (6.8)
Pregnancy history, *n* (%)		
Previous pregnancy	45 (29.4)	51 (34.9)
Previous miscarriage	22 (14.4)	35 (24.0)
Previous still birth	4 (2.6)	1 (0.7)
Previous live birth	27 (17.6)	20 (13.7)
Total ovarian volume, mL		
Mean (SD)	19.0 (50.7)	15.5 (11.8)
Median	12.0	12.6
Basal antral follicles ≥ 5 mm, mean (SD)	n = 153	n = 145
Right ovary	5.3 (3.4)	5.2 (4.3)
Left ovary	5.0 (3.2)	4.8 (4.3)
Basal FSH levels, mU/mL, mean (SD)	7.0 (1.6)	7.3 (2.3)

*ITT* intent-to-treat, *SD* standard deviation, *BMI* body mass index, *FSH* follicle-stimulating hormone

^a^Multiple causes per patient are possible

### Efficacy outcomes

#### Number of oocytes retrieved

At least 1 oocyte was harvested in 295/299 patients in the ITT population (152/153 with Ovaleap® and 143/146 with Gonal-f®). The cancellation rate prior to oocyte retrieval thus was 0.7 % (n = 1) and 2.1 % (n = 3), respectively. In the ATP population, at least 1 oocyte was harvested in 294/297 patients, making the cancellation rate 0.7 % and 1.4 % in the Ovaleap® and Gonal-f® groups, respectively.

The number of oocytes retrieved per patient was similar in both groups. Using an imputation value of zero for patients without oocyte retrieval, Ovaleap®-treated patients had 12.2 ± 6.8 (SD) (median [range]: 11 [0 to 36]) oocytes retrieved vs 11.9 ± 6.9 (median [range]: 12 [0 to 44]) in Gonal-f®–treated patients. Without applying any imputation, oocyte retrievals were nearly identical (12.2 ± 6.7 vs 12.1 ± 6.7, with Ovaleap® and Gonal-f®, respectively). The ZIP regression analysis revealed a mean difference of 0.03 oocytes between treatment groups (Ovaleap® minus Gonal-f®) (95 % CI: –0.76-0.82), which was well within the prespecified equivalence range, thus demonstrating the equivalence of Ovaleap® and Gonal-f®. Results were similar in the ATP population (12.2 ± 6.8 oocytes with Ovaleap® vs 12.0 ± 6.8 oocytes with Gonal-f® [imputed values]), also demonstrating equivalence.

Seventy-five patients in the Ovaleap® group and 61 patients in the Gonal-f® group completed the study without dose adaptation. The mean number of oocytes retrieved in these patients was similar between treatment groups (11.9 vs 12.5). According to the ZIP regression model, age and country had a statistically significant effect on oocyte number (*P* < 0.001) but study treatment did not (*P* = 0.940).

#### r-hFSH dose and duration

Overall, treatment groups were similar with respect to secondary endpoints. Results in the ITT population (described below) were similar to the ATP population (not shown).

The mean total dose of r-hFSH (±SD) was slightly but not significantly lower in the Ovaleap® group compared to the Gonal-f® group (1536 [±496] IU vs 1614 [±485] IU, respectively) (Table 3). The proportions of patients requiring dose adaptations were 51.0 % in the Ovaleap® group and 58.2 % in the Gonal-f® group. In addition, the mean number of days of r-hFSH stimulation was similar in the Ovaleap® group compared with the Gonal-f® group: 9.3 days vs 9.7 days, respectively, with most patients receiving stimulation for 8 to 11 days: 123 (80.4 %) with Ovaleap®, 121 (82.9 %) with Gonal-f®.

**Table 3:** Secondary outcomes related to r-hFSH dosing (ITT population)

Characteristic	Ovaleap® (*n* = 153)	Gonal-f® (*n* = 146)	Odds Ratio (95 % CI)	*P*-value
Total r-hFSH dose, IU, mean (SD)	1536 (496)	1614 (485)	N/A	0.065
Dose adaptation, *n* (%)				
Total	78 (51.0)	85 (58.2)	0.75 (0.47–1.18)	0.215
Dose increase	55 (35.9)	63 (43.2)	0.74 (0.47–1.18)	0.202
Dose decrease	23 (15.0)	22 (15.1)	1.02 (0.52–1.98)	0.964
Duration of r-hFSH stimulation, days				
Mean (SD)	9.3 (1.8)	9.7 (1.6)	N/A	0.131
Median (range)	9 (5–16)	10 (6–16)		

*r-hFSH* recombinant human follicle stimulating hormone, *SD* standard deviation, *N/A* not applicable

#### Follicular, hormonal, and endometrial characteristics

**Fig. 3:**
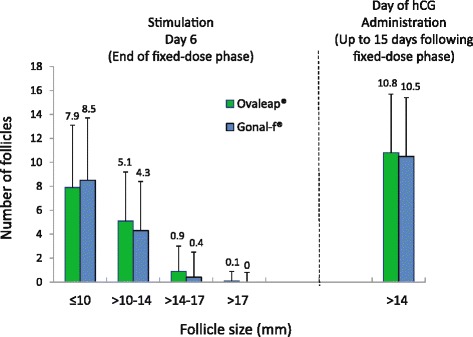
Follicle size after fixed-dose phase (Stimulation Day 6) and follicles >14 mm after dose adaptation (Day of hCG Administration) (ITT population). In Ovaleap® group, n = 153 at both time points; in Gonal-f® group, n = 146 on Day 6 and 144 on hCG day; error bars show standard deviation

There were no clinically relevant differences in the distribution of follicle size at the end of the fixed-dose phase between groups. Both groups had similar substantial increases in the number of follicles > 14 mm at the end of the dose-adaptation phase (Fig. 3). At the end of the fixed-dose phase, the mean number of follicles > 14 mm was 1.0 in the Ovaleap® group and 0.4 in the Gonal-f® group. By the day of hCG administration, the number had increased to 10.8 in the Ovaleap® group and 10.5 in the Gonal-f® group. However, serum estradiol levels were variable. Mean estradiol levels were significantly higher with Ovaleap® than with Gonal-f® at the end of the fixed-dose phase (650.2 vs 516.3 pg/mL; *P* = 0.009) but had increased to similar levels by the day of hCG administration (2744.3 vs 2598.5 pg/mL; *P* = 0.52).

**Fig. 4:**
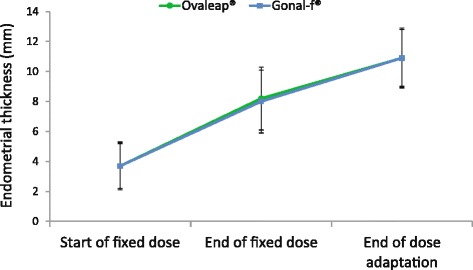
Mean (SD) endometrial thickness over the course of r-hFSH treatment (ITT population)

In both groups, administration of r-hFSH was accompanied by a 3-fold increase in mean endometrial thickness from 3.7 mm at the start of fixed-dose r-hFSH to 10.9 mm on the day of hCG administration that was identical for both groups (Fig. 4). At the end of the fixed-dose phase, endometrial thickness was similar between groups (8.2 mm with Ovaleap® vs 8.0 mm with Gonal-f®).

#### Embryo characteristics and numbers

The morphology of the retrieved 2 PN oocytes was graded as Z1 (best quality) to Z4 (worst quality). Approximately 20 % of the 1689 oocytes were graded as Z1, 40 % as Z2, 30 % as Z3, and 10 % as Z4, with a similar distribution between the treatment groups (ITT population, Table 4).

**Table 4:** Oocyte quality (ITT population)

Characteristic	Ovaleap® (*n* = 144)	Gonal-f® (*n* = 135)
Oocyte quality, n (%) of oocytes		
Z1 (best)	188 (20.7)	164 (21.1)
Z2	337 (37.0)	305 (39.2)
Z3	278 (30.5)	227 (29.1)
Z4 (worst)	107 (11.8)	83 (10.7)
Total 2 PN oocytes	910 (100.0)	779 (100.0)

*ITT* intent-to-treat, *PN* pronucleus

Z score = zygote scoring system of Scott et al. 2000 [12]

The median numbers of embryos obtained and transferred were similar between groups. A median of 3 embryos was obtained per patient in both treatment groups, with a range of 0 to 20 in the Ovaleap®-treated group and a range of 0 to 22 in the Gonal-f® group. A median of 2 embryos (range 0 to 3) was transferred per patient in both treatment groups. In 24 patients (12 in each group), embryo transfers were not performed.

#### Pregnancies

There were no significant differences in clinical and biochemical pregnancy rates between the Ovaleap® and the Gonal-f® groups (Table 5).

**Table 5:** Pregnancy characteristics (ITT population)

	Ovaleap®	Gonal-f®	Odds Ratio (95 % CI)	*P*-value^*^
Biochemical pregnancies				
All patients, n/N (%)	58/153 (37.9)	60/146 (41.1)	0.88 (0.55–1.41)	0.606
Clinical pregnancy rates, n/N (%)				
All patients	43/153 (28.1)	52/146 (35.6)	0.71 (0.43–1.16)	0.172
Patients with oocyte retrieval	43/152 (28.3)	52/143 (36.4)	0.70 (0.42–1.14)	0.150
Patients with embryo transfer	43/141 (30.5)	52/134 (38.8)	0.70 (0.42–1.15)	0.163
Ongoing pregnancy rates, n/N (%)				
All patients	42/153 (27.5)	49/146 (33.6)	0.76 (0.46–1.25)	0.275
Patients with oocyte retrieval	42/152 (27.6)	49/143 (34.3)	0.75 (0.45–1.23)	0.251
Patients with embryo transfer	42/141 (29.8)	49/134 (36.6)	0.74 (0.45–1.24)	0.255
Take-home baby rates, n/N (%)^a^				
All patients	41/153 (26.8)	47/146 (32.2)	0.78 (0.47–1.29)	0.335
Patients with oocyte retrieval	41/152 (27.0)	47/143 (32.9)	0.77 (0.46–1.27)	0.310
Patients with embryo transfer	41/141 (29.1)	47/134 (35.1)	0.77 (0.46–1.28)	0.307

^*^Calculated using a Mantel-Haenszel test, adjusting for the country factor

^a^Take-home baby rate equals the percentage of patients with live births (from fresh or frozen cycles) divided by the number of randomized patients. All other outcomes reported in this table only include pregnancies resulting from fresh cycles

Fifty-eight patients (37.9 %) in the Ovaleap® group had biochemical pregnancies compared with 60 (41.1 %) in the Gonal-f® group (*P* = 0.606). Clinical pregnancy rates were 28.1 % (43/153) in the Ovaleap® group and 35.6 % (52/146) in the Gonal-f® group (*P* = 0.172). In addition, clinical pregnancy rates with Ovaleap® and Gonal-f® were similar among patients who had oocytes retrieved and similar among patients who underwent embryo transfer.

However, a post-hoc analysis revealed substantial variability in clinical pregnancy rates from country to country (28.6 % [10/35] with Ovaleap® and 16.7 % [5/30] with Gonal-f® in Belgium-Germany, 30.0 % [9/30] with Ovaleap® and 39.3 % [11/28] with Gonal-f® in the Czech Republic, 35.9 % [14/39] with Ovaleap® and 44.7 % [17/38] with Gonal-f® in Hungary, and 20.4 % [10/49] with Ovaleap® and 38.0 % [19/50] with Gonal-f® in Poland) and from center to center. An additional 4 patients who did not achieve biochemical pregnancy after embryo transfer received their frozen embryos obtained during this study and became pregnant. Thus, a total of 99 pregnancies were recorded (46 Ovaleap® and 53 Gonal-f®).

Rates of ongoing pregnancies, defined by ultrasound (or by birth or stillbirth if ultrasound information was missing), were 27.5 % for patients treated with Ovaleap® and 33.6 % with Gonal-f® (Table 5). A post-hoc analysis revealed variability in ongoing pregnancy rates from country to country (25.7 % [9/35] with Ovaleap® and 13.3 % [4/30] with Gonal-f® in Belgium-Germany, 23.3 % [7/30] with Ovaleap® and 32.1 % [9/28] with Gonal-f® in the Czech Republic, 35.9 % [14/39] with Ovaleap® and 44.7 % [17/38] with Gonal-f® in Hungary, and 24.5 % [12/49] with Ovaleap® and 38.0 % [19/50] with Gonal-f® in Poland) and from center to center as seen in the post-hoc analysis of clinical pregnancy rates.

The incidences of pregnancy loss were similar in both groups, occurring in 8/153 (5.2 %) of Ovaleap®-treated patients and 9/146 (6.2 %) of Gonal-f®–treated patients. Ectopic pregnancy occurred in 2 Ovaleap®-treated patients (1.3 %) and in 1 Gonal-f®–treated patient (0.7 %); none were assessed by the investigator as related to study medication. Three patients had other complications, including Down’s syndrome (Ovaleap® group [with subsequent clinical abortion]), premature delivery of triplets (Gonal-f® group), and epilepsia paroxysm (Gonal-f® group).

#### Live births and take-home baby rates

Approximately 90 % of clinically pregnant patients went on to have a live birth, 89.1 % (41/46) in the Ovaleap® group and 88.7 % (47/53) in the Gonal-f® group. Take-home baby rates, defined as the percentage of randomized patients with live births, were 26.8 % (41/153) with Ovaleap® and 32.2 % (47/146) with Gonal-f® (Table 5). Overall, a total of 111 live births occurred in the study and were similarly distributed between the 2 treatment groups, with 54 live babies in the Ovaleap® group and 57 live babies in the Gonal-f® group. Multiple births occurred in 22 women, or 25 % of the participants who had live births. In the Ovaleap® group, 13/41 patients who had live births (31.7 %) had twins. There were 8 patients with twins and 1 patient with triplets in the Gonal-f® group (9/47 live births, 19.1 %). Two babies born prematurely to women in the Gonal-f® group died (1 singleton, 1 of a set of triplets). One patient in the Gonal-f® group had a still birth, and no still births occurred in the Ovaleap® group.

#### Safety and tolerability

Overall frequencies of TEAEs were low and comparable across treatment groups: 16.3 % (25/153) in the Ovaleap® group and 15.1 % (22/146) in the Gonal-f® group. The most common TEAEs were OHSS (4.6 % [7/153] in the Ovaleap® group and 2.7 % [4/146] in the Gonal-f® group), abdominal pain (3.3 % [5/153] Ovaleap®; 0.7 % [1/146] Gonal-f®), and missed abortion (2.1 % [3/146] Gonal-f®) (Table 6). Severe TEAEs were reported in 7 patients (3 Ovaleap®, 4 Gonal-f®) and included 2 events of OHSS (1 per treatment group).

**Table 6:** TEAEs occurring in 2 or more patients during the main study, n (%) (Safety Population)

Symptom	Ovaleap® (*n* = 153)	Gonal-f® (*n* = 146)	Total (*N* = 299)
OHSS	7 (4.6)	4 (2.7)	11 (3.7)*
Abdominal pain	5 (3.3)	1 (0.7)	6 (2.0)
Abortion missed	0	3 (2.1)	3 (1.0)
Ectopic pregnancy	2 (1.3)	1 (0.7)	3 (1.0)**
Nasopharyngitis	1 (0.7)	2 (1.4)	3 (1.0)
Nausea	2 (1.3)	1 (0.7)	3 (1.0)
Antepartum hemorrhage	1 (0.7)	1 (0.7)	2 (0.7)
Headache	2 (1.3)	0	2 (0.7)

OHSS, ovarian hyperstimulation syndrome; TEAEs, treatment-emergent adverse events

*P = 0.54

**P = 1.0

OHSS was reported in 11 patients (7 Ovaleap® [4.6 %]; 4 Gonal-f® [2.7 %]). The difference in the frequency of OHSS between groups was not statistically significant (*P* = 0.542). OHSS was rated as mild in 3 patients assigned to Ovaleap® and 2 patients assigned to Gonal-f®, moderate in 3 patients assigned to Ovaleap® and 1 patient assigned to Gonal-f®, and severe in 1 patient in each group. No cases of life-threatening OHSS were reported; however, OHSS led to hospitalization in 1 patient in each group. OHSS led to treatment discontinuation in 1 patient in the Ovaleap® group and 2 patients in the Gonal-f® group. All cases had resolved by the end of the study, except 1 case in which the outcome is unknown.

Serious TEAEs occurred in 16 patients, including abortion (2 Ovaleap®, 3 Gonal-f®), OHSS (3 Ovaleap®, 2 Gonal-f®), ectopic pregnancy (2 Ovaleap®, 1 Gonal-f®), abdominal pain (1 each group), and antepartum hemorrhage (Ovaleap®). Of the 17 pregnancy losses that occurred during the study, 10 were not considered TEAEs since they occurred > 30 days after completion of treatment with r-hFSH.

There were no clinically relevant changes in laboratory variables, electrocardiogram, physical examination, body weight, or vital signs that emerged as a result of treatment. Nonneutralizing antibodies toward of IgA, IgG, and IgM classes were detected in the immunogenicity assays but were not clinically relevant because they were predominantly directed at motifs not present in CHO cell-produced glycoproteins. Among the 22 patients who showed post-dose positive findings using the revised highly sensitive assay, 5 treated with Gonal-f® and 11 treated with Ovaleap® in the first treatment cycle had positive findings, which were mainly against Neu5Gc. However, none of these positive findings showed neutralizing activity. No positive findings related to the IgE class were identified.

#### Patient-reported outcomes

Overall, tolerability was good and comparable between patient groups, with minimal injection-site pain and injection-site reactions.

Patients were highly satisfied with both Ovaleap® and Gonal-f® pen devices (Table 7); > 75 % in both groups gave a rating of “very confident” in terms of accurate dosing and correct injection and “very convenient” and “very satisfied” in terms of administration. More than 70 % rated the instructional text to be “very easy” to understand. Less than 5 % needed more than 1 explanation about how to use the device.

**Table 7:** Patient satisfaction with study pen device, n (%)

Characteristic	Ovaleap® (*n* = 151)	Gonal-f® (*n* = 142)
Confidence about accurate dose		
Very confident	123 (81.5)	112 (78.9)
Confident	28 (18.5)	30 (21.1)
Confidence about correct injection		
Very confident	115 (76.2)	110 (77.5)
Confident	34 (22.5)	32 (22.5)
Not confident	1 (0.7)	0 (0)
Plainness of instructional text		
Very easy	113 (74.8)	100 (70.4)
Easy	38 (25.2)	42 (29.6)
Frequency of need for explanation of administration		
Never	85 (56.3)	67 (47.2)
Once	57 (37.7)	69 (48.6)
Twice	7 (4.6)	2 (1.4)
Three times	0 (0)	1 (0.7)
Convenience of pen usage		
Very convenient	117 (77.5)	107 (75.4)
Convenient	34 (22.5)	35 (24.6)
Satisfaction with administration		
Very satisfied	116 (76.8)	108 (76.1)
Satisfied	35 (23.2)	34 (23.9)

## Discussion

Our phase 3 patient-study found Ovaleap® to be equivalent to Gonal-f® in the primary endpoint of the number of oocytes retrieved in a population of infertile women undergoing ovulation stimulation during ART, which is the primary endpoint recommended by the EMA for trials comparing Gonal-f® to biosimilar preparations. In addition, the secondary study endpoints of follicle number and size and endometrial thickness were comparable between groups at the end of the fixed-dose phase, and the numbers of oocytes retrieved were similar in patients who did not require dose adaptation, suggesting the clear comparability of these formulations. Oocyte quality, as assessed by Z scores, was also similar between groups, with no clinically significant differences observed. The number of oocytes retrieved in patients treated with Ovaleap® and Gonal-f® were also comparable to those in previous studies of Gonal-f® in infertile patients who were treated with a long protocol and down-regulated with a GnRH agonist [15–20].

The clinical pregnancy rate for patients associated with Gonal-f® treatment has ranged from 23 % to 39 % in the literature [15, 17]. The clinical pregnancy rates with embryo transfer of 30.7 % for Ovaleap®-treated patients and 38.8 % for Gonal-f®–treated patients in this study both are consistent with that range. The difference in clinical pregnancy rates between groups was not statistically significant and may be indicative of the substantial variability in clinical pregnancy rates we observed between study sites and countries of origin. For example, clinical pregnancy rates were as low as 20.4 % with Ovaleap® in Poland and as high as 35.9 % in Hungary. With Gonal-f®, clinical pregnancy rates were as low as 16.7 % in Belgium + Germany and as high as 44.7 % in Hungary.

Higher clinical pregnancy rates in the Gonal-f® group were seen in the Czech Republic (42.3 % vs 36.0 %), Hungary (46.0 % vs 36.8 %), and Poland (40.4 % vs 20.8 %). In Belgium + Germany, however, the clinical pregnancy rate was higher in the Ovaleap® group than in the Gonal-f® group (33.3 % vs 20.8 %). The stratified post-hoc analysis of clinical and ongoing pregnancy rates by baseline and post-baseline characteristics did not reveal any findings to explain the differences in rates among sites and countries.

An important goal toward improving ART is to lower the rate at which couples discontinue ART. Interviews with couples have revealed that the emotional stress or psychological burden involved with the treatment process is an important factor determining the risk of treatment discontinuation [21, 22]. Research with regard to patient preferences during infertility treatment is scarce; although patient preference or perceived convenience may drive the choice of gonadotropic agent. For example, a recent questionnaire study conducted in Sweden showed that patients preferred ovarian stimulation treatments that they believed would reduce dose variability and were easy to use [23]. The authors of this study speculated that patients equate dose variability with poor quality and a greater risk of TEAEs, such as OHSS.

In the present study, the Ovaleap® pen device received high patient ratings in overall ease of use and confidence in accurate dosing. Patients were able to self-administer after only one demonstration on site. Although the majority of patients had previous experience using the Gonal-f® pen, patients assigned to Ovaleap® were similarly confident about the accuracy of dosing and administration, indicating the ease of use of the Ovaleap® pen. Taking patient convenience into consideration, using pen devices may be an important way to help reduce the psychological stress of fertility treatment and perhaps improve treatment success [24].

Ovaleap® was found to have a favorable safety profile, similar to that of Gonal-f®, with no new unexpected safety concerns identified. OHSS was the most common TEAE (11/153, 7.2 %), and all episodes (except in 1 patient who was lost to follow-up) resolved. The numbers of OHSS cases were small with no statistically significant differences between groups, and comparable to that seen with other FSH products [11, 18, 25–27].

One potential limitation of this randomized, multicenter, and otherwise well-designed study is that the lack of patient blinding due to the use of the patent-protected Gonal-f® pen device could result in bias. However, because this study design was similar to that of other comparative studies of ART using r-hFSH [18, 19], this potential bias was not considered to be a significant concern. It is also conceivable that regional variations in laws, practices, and preferences with regard to ART practices, which may have contributed to the differences in clinical and ongoing pregnancy rates from country to country, may have introduced bias. In addition, the relative leanness of the patients included in this study (mean BMI, 22 kg/m^2^) should also be considered when generalizing study results to other patient populations.

Although the primary endpoint of our study was the number of oocytes retrieved, live birth rates might have been a more clinically meaningful outcome to patients. However, the number of oocytes retrieved is the primary endpoint recommended by the European Medicines Agency for studies evaluating the clinical comparability of Gonal-f® and biosimilar formulations [5], and using live birth rates as a primary outcome would have required a much larger, more resource-intensive study. In addition, pregnancy rates and live birth rates are influenced by a variety of factors beyond the scope of r-hFSH treatment that are difficult to control. Moreover, a strong association between the number of retrieved eggs and live birth rates has previously been documented [10].

## Conclusion

We found Ovaleap® to be a safe and effective r-hFSH for stimulation of follicular development in infertile women undergoing ART and similar to Gonal-f® in its safety and efficacy. The availability of a new r-hFSH product will give patients and providers another treatment option, increasing the accessibility and possibly reducing the stress associated with ART. An additional post-authorization, multinational, multicenter, prospective, observational cohort study will further evaluate the use of Ovaleap® in patients seeking treatment for infertility [25].

## Abbreviations

ART: assisted reproductive technology
ATP: according-to-protocol
AUC_0-t_: area under the concentration vs time curve
BMI: body mass index
CHO: Chinese hamster ovary
C_max_: peak plasma concentration
ECG: electrocardiogram
FSH: follicle-stimulating hormone
hCG: human chorionic gonadotropin
hMG: human menopausal gonadotropin
ITT: intent-to-treat
IVF: in vitro fertilization
OHSS: ovarian hyperstimulation syndrome
PN: pronucleus
r-hFSH: recombinant human follicle-stimulating hormone
SC: subcutaneous
TEAE: treatment-emergent adverse event
ZIP: zero-inflated Poisson regression

## References

[1] De Leo V, Musacchio MC, Di Sabatino A, Tosti C, Morgante G, Petraglia F (2012). Present and future of recombinant gonadotropins in reproductive medicine. Curr Pharm Biotechnol.

[2] Leao RB, Esteves SC (2014). Gonadotropin therapy in assisted reproduction: an evolutionary perspective from biologics to biotech. Clinics.

[3] Daya S (2004). Follicle-stimulating hormone in clinical practice: an update. Treat Endocrinol.

[4] Harrison S, Wolf T, Abuzeid MI (2000). Administration of recombinant follicle stimulating hormone in a woman with allergic reaction to menotropin: a case report. Gynecol Endocrinol.

[5] Committee for Medicinal Products for Human Use (CHMP) (2011). Guideline on non-clinical and clinical development of similar biological medicinal products containing recombinant human follicle stimulating hormone (r-hFSH).

[6] Weise M, Bielsky MC, De Smet K, Ehmann F, Ekman N, Narayanan G (2011). Biosimilars-why terminology matters. Nat Biotechnol.

[7] Lammerich A, Bias P, Gertz B (2015). Phase 1 safety, tolerability, and pharmacokinetic study of single ascending doses of XM17 (recombinant human follicle-stimulating hormone) in downregulated healthy women. Int J Womens Health.

[8] Lammerich A, Mueller A, Bias P (2015). Phase I, two-way, crossover study to demonstrate bioequivalence and to compare safety and tolerability of single-dose XM17 vs Gonal-f® in healthy women after follicle-stimulating hormone downregulation. Reprod Biol Endocrinol.

[9] Gertz B, Lammerich A. Phase I Study to establish the bioequivalence of recombinant human follicle stimulating hormone (r-hFSH; XM17) to Gonal-f® (follitropin alfa) in downregulated healthy female subjects [abstract]. Presented at: 10th Congress of the European Society of Gynecology, September 18-21, 2013, Brussels, Belgium.

[10] Sunkara SK, Rittenberg V, Raine-Fenning N, Bhattacharya S, Zamora J, Coomarasamy A (2011). Association between the number of eggs and live birth in IVF treatment: an analysis of 400 135 treatment cycles. Hum Reprod.

[11] Papanikolaou EG, Pozzobon C, Kolibianakis EM, Camus M, Tournaye H, Fatemi HM (2006). Incidence and prediction of ovarian hyperstimulation syndrome in women undergoing gonadotropin-releasing hormone antagonist in vitro fertilization cycles. Fertil Steril.

[12] Scott L, Alvero R, Leondires M, Miller B (2000). The morphology of human pronuclear embryos is positively related to blastocyst development and implantation. Hum Reprod.

[13] Somkuti SG, Schertz JC, Moore M, Ferrande L, Kelly E (2006). Patient experience with follitropin alfa prefilled pen versus previously used injectable gonadotropins for ovulation induction in oligoanovulatory women. Curr Med Res Opin.

[14] Bergh C, Howles CM, Borg K, Hamberger L, Josefsson B, Nilsson L (1997). Recombinant human follicle stimulating hormone (r-hFSH; Gonal-F) versus highly purified urinary FSH (Metrodin HP): results of a randomized comparative study in women undergoing assisted reproductive techniques. Hum Reprod.

[15] Frydman R, Howles CM, Truong F (2000). A double-blind, randomized study to compare recombinant human follicle stimulating hormone (FSH; Gonal-F®) with highly purified urinary FSH (Metrodin® HP) in women undergoing assisted reproductive techniques including intracytoplasmic sperm injection. Hum Reprod.

[16] European and Israeli Study Group on Highly Purified Menotropin versus Recombinant Follicle-Stimulating Hormone. Efficacy and safety of highly purified menotropin versus recombinant follicle-stimulating hormone in in vitro fertilization/intracytoplasmic sperm injection cycles: a randomized, comparative trial. Fertil Steril*.* 2002;78:520-8. 10.1016/s0015-0282(02)03250-8 12215327

[17] Westergaard LG, Erb K, Laursen SB, Rex S, Rasmussen PE (2001). Human menopausal gonadotropin versus recombinant follicle-stimulating hormone in normogonadotropic women down-regulated with a gonadotropin-releasing hormone agonist who were undergoing in vitro fertilization and intracytoplasmic sperm injection: a prospective randomized study. Fertil Steril.

[18] Schats R, Sutter PD, Bassil S, Kremer JA, Tournaye H, Donnez J (2000). Ovarian stimulation during assisted reproduction treatment: a comparison of recombinant and highly purified urinary human FSH. Hum Reprod.

[19] Andersen AN, Devroey P, Arce JC (2006). Clinical outcome following stimulation with highly purified hMG or recombinant FSH in patients undergoing IVF: a randomized assessor-blind controlled trial. Hum Reprod.

[20] Rettenbacher M, Andersen AN, Garcia-Velasco JA, Sator M, Barri P, Lindenberg S (2015). A multi-centre phase 3 study comparing efficacy and safety of Bemfola® versus Gonal-f® in women undergoing ovarian stimulation for IVF. Reprod Biomed Online.

[21] Verberg MF, Eijkemans MJ, Heijnen EM, Broekmans FJ, de Klerk C, Fauser BC (2008). Why do couples dropout from IVF treatment? A prospective cohort study. Hum Reprod.

[22] Brandes M, van der Steen JO, Bokdam SB, Hamilton CJ, de Bruin JP, Nelen WL (2009). When and why do subfertile couples discontinue their fertility care? A longitudinal cohort study in a secondary care subfertility population. Hum Reprod.

[23] Landfeldt E, Jablonowska B, Norlander E, Persdotter-Eberg K, Thurin-Kjellberg A, Wramsby M (2012). Patient preferences for characteristics differentiating ovarian stimulation treatments. Hum Reprod.

[24] Boivin J, Domar AD, Shapiro DB, Wischmann TH, Fauser BC, Verhaak C (2012). Tackling burden in ART: an integrated approach for medical staff. Hum Reprod.

[25] European Medicines Agency. Ovaleap (follitropin alfa) [assessment report]. http://www.ema.europa.eu/docs/en_GB/document_library/EPAR_-_Public_assessment_report/human/002608/WC500152908.pdf (2013). Accessed February 25, 2015.

[26] Jayaprakasan K, Hopkisson J, Campbell B, Johnson I, Thornton J, Raine-Fenning N (2010). A randomised controlled trial of 300 versus 225 IU recombinant FSH for ovarian stimulation in predicted normal responders by antral follicle count. BJOG.

[27] Latin-American Puregon IVF Study Group (2001). A double-blind clinical trial comparing a fixed daily dose of 150 and 250 IU of recombinant follicle-stimulating hormone in women undergoing in vitro fertilization. Fertil Steril.

